# Ammonio-sulphate of Copper

**Published:** 1853-03

**Authors:** 


*Ammonio-sulphate of Copper.*—At the Westminster Hospital, Dr. Hamilton Roe has recently employed this salt very successfully in the treatment of a series of cases of severe cholera, the patients being mostly children under fourteen years of age. In several the sulphate of zinc and sesquichloride of iron had been tried previously, and without benefit. The dose given has been from half a grain to a grain three times daily. Its use in this complaint is not novel, although not much resorted to in this country. In Italy it has gained a considerable reputation, and is known by the name of the "specific of Stissero." Mr. Startin has for some time been accustomed to prescribe a long continued course of the ammoniated copper in malignant diseases, and seemingly with some good results. Its action is probably that of a tonic. In several cases the health of the patient has appeared to improve very perceptibly. Scirrhus of the breast and cancroid ulcers of the skin are the forms in which it has principally been tried. The dose has ranged from the eighth to a quarter of a grain three times daily, and has often been continued for many months. We believe it has been employed for nearly similar purposes in veterinary medicine.—*Ibid.*

